# Assessment of Position Repeatability Error in an Electromagnetic Tracking System for Surgical Navigation

**DOI:** 10.3390/s20040961

**Published:** 2020-02-11

**Authors:** Gregorio Andria, Filippo Attivissimo, Attilio Di Nisio, Anna Maria Lucia Lanzolla, Mattia Alessandro Ragolia

**Affiliations:** Department of Electrical and Information Engineering, Polytechnic University of Bari, Bari, BA, 70126, Italy; gregorio.andria@poliba.it (G.A.); attilio.dinisio@poliba.it (A.D.N.); anna.lanzolla@poliba.it (A.M.L.L.); mattiaalessandro.ragolia@poliba.it (M.A.R.)

**Keywords:** electromagnetic tracking system, image guided surgery, surgical navigation, position repeatability error, error propagation

## Abstract

In this paper we present a study of the repeatability of an innovative electromagnetic tracking system (EMTS) for surgical navigation, developed to overcome the state of the art of current commercial systems, allowing for the placement of the magnetic field generator far from the operating table. Previous studies led to the development of a preliminary EMTS prototype. Several hardware improvements are described, which result in noise reduction in both signal generation and the measurement process, as shown by experimental tests. The analysis of experimental results has highlighted the presence of drift in voltage components, whose effect has been quantified and related to the variation of the sensor position. Repeatability in the sensor position measurement is evaluated by means of the propagation of the voltage repeatability error, and the results are compared with the performance of the Aurora system (which represents the state of the art for EMTS for surgical navigation), showing a repeatability error about ten times lower. Finally, the proposed improvements aim to overcome the limited operating distance between the field generator and electromagnetic (EM) sensors provided by commercial EM tracking systems for surgical applications and seem to provide a not negligible technological advantage.

## 1. Introduction

Minimally invasive surgery (MIS) includes techniques that are designed to reduce the trauma associated with surgical interventions [1,2,3,4]. Executing fewer and smaller incisions has numerous benefits to the patient, including reduced risk of infection and post-operative pain, faster recovery time and minimal scarring.

Significant support is provided by Computer Assisted Surgery (CAS), which relies on medical imaging techniques such as Magnetic Resonance Imaging (MRI) and Computed Tomography (CT), Ultrasound (US) to provide a three-dimensional model of the patient’s anatomy [5,6,7,8,9,10,11,12,13], allowing for diagnostic and the pre-operative planning of interventions. CAS combines the anatomical model with the real-time position and orientation of surgical instruments, provided by tracking systems and guiding the surgeon during the intervention.

Electromagnetic tracking systems (EMTSs) are widely used in surgery navigation because they do not require a direct line of sight between the sensors and the field generator, unlike the optical tracking systems, and offer the possibility of using very small magnetic sensors, inserted into the surgical instruments, to measure the magnetic fields of known geometry, generated by a field source [14,15,16]. The sensor signal is then used to determine the position and the orientation of the surgical tools in the operative scenario.

On the other hand, EMTS technology presents two main limitations: (a) it has high sensitivity to EM interferences provided by electronic devices and to magnetic field distortions due to metal objects; (b) current commercial systems are not able to provide accurate position estimations over a large distance from the signal source, due to the degradation of the magnetic field amplitude with the distance from the field generator. Hence, the signal source must be placed near the operating table, hindering the medical staff during the operation. Typically, the tracking volume is located about 0.5 m from the signal source when using sensors with a diameter smaller than 1 mm, that are needed for tracking surgical instruments such as endoscopes and needles [16,17]. 

In this paper we describe a performance analysis technique applied to an innovative EMTS, developed to overcome the state of the art of current commercial systems, allowing for the placement of the signal source beyond 0.5 m from the operating table. The paper is structured as follows.

In Section 2, an overview of the operating principle of the EMTS prototype is given, and the experimental setup is shown. In Section 3, several improvements performed on the system are described. Their effect on the system’s performance is analyzed in Section 4 by evaluating the repeatability of the sensor’s signal, and a careful analysis of the voltage drift is performed. In Section 5, a method based on error propagation is proposed for the assessment of the position error, evaluating the position error due to sensor repeatability and drift errors, and a comparison with the state of the art is made. Finally, conclusions are drawn in Section 6.

## 2. Electromagnetic Tracking System (EMTS) Overview

**Figure 1:**
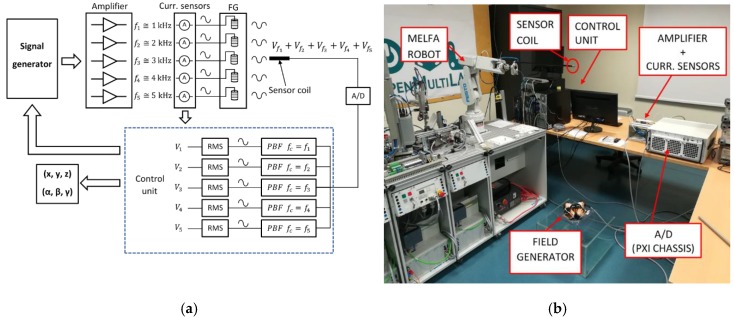
(**a**) Electromagnetic tracking system (EMTS) architecture; the signals produced by a signal generator are amplified, providing the excitation currents for the five transmitting coils. The total magnetic field induces a voltage in the sensor coil, which is digitalized and filtered, obtaining five RMS voltage components, $V_{i},\;i=1,\;\ldots ,\;5$, relating to the different excitation frequencies. These components are then used for the estimation of the sensor position. Moreover, five Hall effect sensors measure the excitation currents for the current control loop. (**b**) Experimental setup.

The EMTS prototype has been developed in MASMEC Biomed company (Italy), in collaboration with the Polytechnic University of Bari (Italy), to overcome the limitations of current commercial systems. The system has been carefully presented and characterized in [18,19,20], where the position sensor was reconstructed using triangulation and the piecewise linear interpolation technique. It is composed of three main components: a field generator (FG), a small sensor coil (Aurora – NDI) [21] and a control unit, as shown in Figure 1a.

The FG is composed of five transmitting coils, arranged in space to minimize mutual coupling effects, which have been quantified in [20]. The transmitting coils are powered by sinusoidal currents at different frequencies (approximately 1, 2, 3, 4 and 5 kHz), according to the Frequency Division Multiplexing (FDM) technique. FDM enhances the use of time resources and does not require a synchronization device, unlike Time Division Multiplexing (TDM).

According to the IEEE Standard C95.1-2005 [22,23], which defines the security levels for human exposure to electromagnetic fields, the amplitude of the total generated magnetic field must not exceed the limit value of 0.2 mT. Hence, excitation currents in each coil were limited to 1 A rms, as detailed in [18]. In this way, the threshold of 0.2 mT was respected from a distance of about 15 cm from the center of the field generator.

The FG provided an induced voltage on the magnetic sensor (MS) that included the contribution of each generated magnetic field; then, the five voltage components were extracted, obtaining five rms voltage components, as shown in Figure 1a. They were used to estimate the position of the sensor by means of a suitable reconstruction algorithm.

In Figure 1b, the experimental setup is shown. It consists of the FG prototype, the coil sensor, the MITSUBISHI MELFA robot [24], with a position repeatability of 0.02 mm for the movement of the sensor, and the data acquisition (DAQ) system, based on PXI (PCI eXtensions for Instrumentation) technology.

The control software developed in LabVIEW® performs automatic tests and measurements, and provides real-time parameters to monitor the system state. Moreover, it allows the storing of the data sets for subsequent offline processing for performance analysis [25,26,27].

## 3. Hardware Development

The experimental setup described in [20] has been carefully examined, leading to new developments, in order to reduce the measurement noise.

### 3.1. DAQ Synchronization

In [20], the impact of spectral leakage errors has been shown, and remedial actions have been performed. However, by analyzing the spectrum of the currents flowing in the transmitting coils, spectral leakage was still observed. This phenomenon was also observed in the time domain: the rms current value was affected by a periodic oscillation, whose frequency increased with signal frequency. A careful analysis has led us to identify this error as a result of non-coherent sampling. Indeed, even though the signal frequencies were chosen according to their coherent sampling condition, the DAQ device was not able to sample at the proper frequency, due to its finite resolution, causing a frequency mismatch. Moreover, there was a clock mismatch between the generation and the acquisition devices. Hence, we finely tuned the sampling frequency, still maintaining the coherent sampling condition, and synchronized the time bases of the generation and acquisition devices through the routing of the clock signal. Finally, we set the sampling frequency $f_{s}=50\;\mathrm{kHz}$ and the number of samples per channel $N_{s}=2500$. The results show an improved field stability of more than 4 times, with standard deviations of about 0.007% of the FG current of 1 A rms.

### 3.2. Analysis of the Electronic Board

The NI PXIe-1065 (National Instrument, Austin, TX, USA) chassis has been configured with two boards for the generation and acquisition of signals: the analog output device produces five signals, which are amplified by five OPA 544 (Analog Devices, Norwood, MA, USA) and power the transmitting coils; the analog input device is used to acquire the signal from the coil sensor and the five transmitting current signals by means of Hall effect sensors LA 55-P (LEM International SA, Plan-les-Ouates, Switzerland).

Experimental results have highlighted a conductive coupling effect due to common power and ground returns, adding noise to the measurement of the sensor’s induced voltage. To avoid this effect, separated power and ground returns have been provided, reducing the voltage noise by more than 6 dB.

### 3.3. Improved DAQ Devices

In the previous experimental setup, a NI PXI-6052E (National Instrument, Austin, TX, USA) device with 16-bit resolution and low noise was used to acquire both the sensor voltage and the current sensors signal. Due to the low amplitude of induced voltage, an INA 114 was used to amplify the signal.

Experimental tests have shown that the instrumentation amplifier provides noise in the measurement process. In order to reduce this effect, a 24-bit DAQ device (NI PXI-4461) (National Instrument, Austin, TX, USA) was used for the acquisition of the voltage induced on the coil sensor, allowing for the removal of the amplifier. It presented an input range of ±0.316 V_pk_ at 30 dB gain, with an LSB of about 38 nV, more than 80 times lower than the PXI-6052E.

### 3.4. Cable’s Shielding

With the aim of evaluating the noise floor of the acquiring device, the voltage measured on the sensor with the FG turned off was also acquired. 

In this case, the voltage noise is mainly due to Johnson noise having power spectral density (PSD) given by 4kTR. By considering T = 296 K and R = 80 Ω (corresponding to the resistance of sensor coil), noise power densisty is −179 dB/Hz, or 1.14 $\mathrm{nV}/\sqrt{\mathrm{Hz}}$.

The noise PSD of the PXI-4461 is −162 dB/Hz, or 8 $\mathrm{nV}/\sqrt{\mathrm{Hz}}$. Therefore, when the sensor is connected to the analog input channel with FG turned off, a noise floor of about −162 dB/Hz should be measured. Instead, experimental results showed a value of about −145 dB/Hz. Consequently, there was an error source that increased the measurement noise.

In order to reduce noise, proper shielding was considered. Careful experimentation showed that using a shielded cable of about 2 m to connect the coil sensor to the DAQ device, the best results are obtained by connecting the cable shield to the chassis ground with a short wire, taking care to make the path as short as possible. This has allowed to reduce the noise floor to −162 dB/Hz, as expected.

## 4. Performance Analysis

### 4.1. Induced Voltage Repeatability

To analyze the effect of the improvements discussed in Section 3, the same experimental tests executed in [20] have been performed, and the obtained results have been compared.

**Figure 2:**
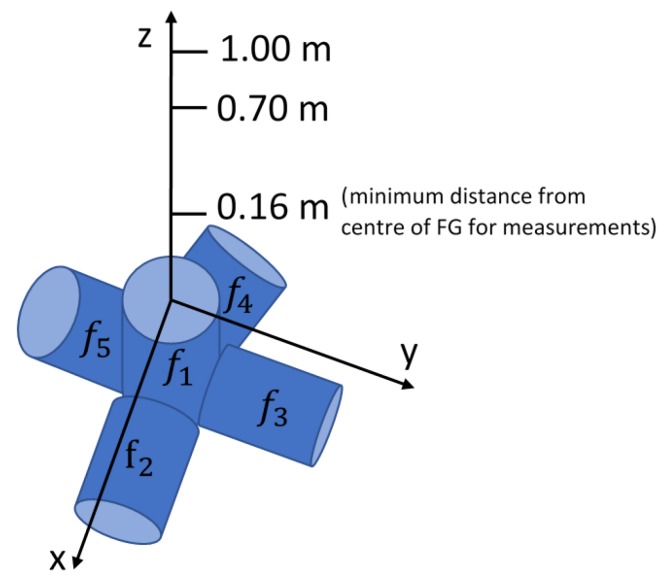
Coordinate system with reference to the field generator (FG).

The FG was supplied with currents of 1 A rms for each channel, and two baselines for sensor positioning were defined, respectively at 0.7 m and 1 m from the FG, along the *z*-axis of the reference system (Figure 2). For each position, 200 measurements of the five rms voltage components $V_{i}$, as well as the rms currents $I_{i}$, i=1,…, 5, were executed, and the mean $\bar{V_{i}}$ and standard deviation (SD) $\sigma_{v}{}_{i}$ were calculated. The sampling frequency, $f_{s}=50\;\mathrm{kHz}$, was selected, and $N_{s}=2500$ samples were acquired; therefore, the rms values were updated with a frequency $f_{up}=\frac{f_{s}}{N_{s}}=20\;\mathrm{Hz}$, which is the minimum rate appropriate for surgical navigation. The measurements have been performed keeping the sensor oriented along the *x*-axis, parallel to the coil powered at $f_{2}$.

Since the INA 114 instrumentation amplifier has been removed in the new hardware development, the magnitudes of the induced voltages were different. Hence, to correctly compare the effect of hardware changes, the relative SD values were considered, defined as:

(1) $$\sigma_{v,rel}{}_{i}=\frac{\sigma_{v}{}_{i}}{\bar{V_{i}}}$$

Table 1 lists the relative SD of the induced voltage, at 0.7 m and 1 m, obtained before and after the hardware improvement. An overall noise reduction of about 70% for almost all channels can be observed. Moreover, it can be observed that some channels present a relative SD significantly lower than others. In particular, the first and third channels present higher values, due to the coupling between the sensor coil and each transmitting coil, which depends on sensor orientation and transmitting coils arrangement, which affect mutual induction and the concatenated magnetic flux.

### 4.2. Induced Voltage Drift

The current which flows in each transmitting coil causes the heating of the coil, with consequent variations of coils resistance which result in magnetic field amplitude variations. Hence, for each coil, a current control loop has been implemented [20], to keep the magnetic field amplitude stable over time. The control system is based on the use of the five Hall effect sensors LA 55-P, to measure the current in each transmitting coil.

**Figure 3:**
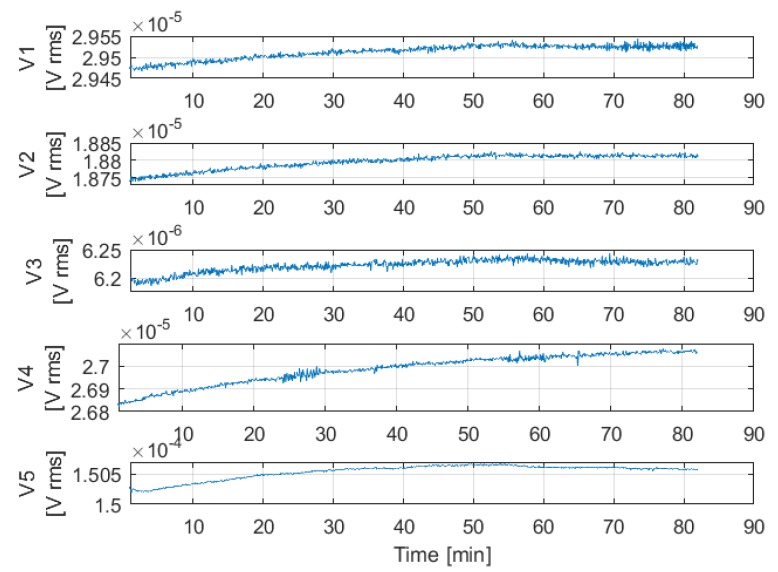
Voltage drift vs time; current loop turned on.

Despite the current control system, experiments have shown a significant drift of voltage components, as shown in Figure 3, representing the voltage values acquired in 82 min, after a system warm-up of about 30 min, with the sensor placed in a fixed position at 0.7 m along the *z*-axis.

It can be noticed that, after about 50 min, the first three channels seem to converge, whereas the fifth starts to decrease and the fourth channel is still increasing. Therefore, it is not possible to predict how the drift will affect the system.

To investigate the cause of the drift phenomena, additional voltage acquisitions have been performed. The current control loop was turned on, employing $I_{i}$ as regulated variable [Appendix A], and only one channel has been powered with a current of 1 A rms, keeping the other channels turned off. A multimeter, model HP 34401A, has been inserted in series with the active transmitting coil, to provide a reference measurement of the excitation current. The acquisition started after a zero warm-up time.

**Figure 4:**
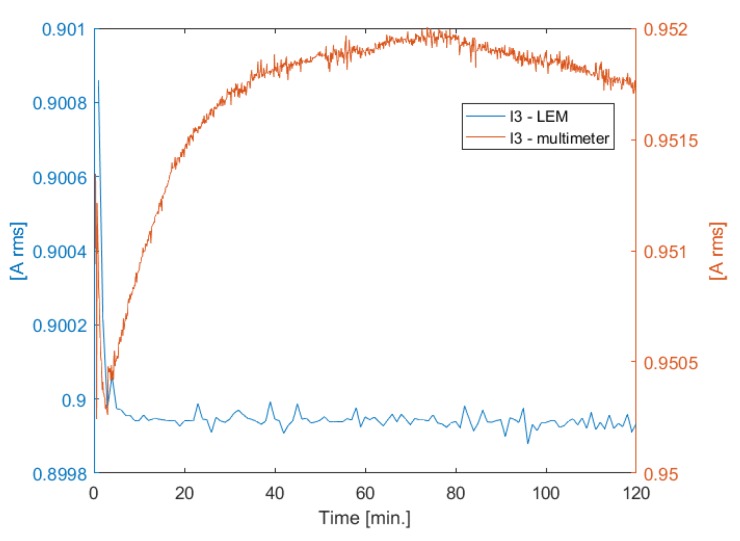
Current measured with a Hall effect sensor and with a multimeter—channel 3.

Figure 4 shows the comparison between the current measured with the Hall effect sensor and with the multimeter, related to the third transmitting coil. We can observe that the current measured with the Hall effect sensor converges after a very short time, while the current measured with the multimeter drifts over time.

**Figure 5:**
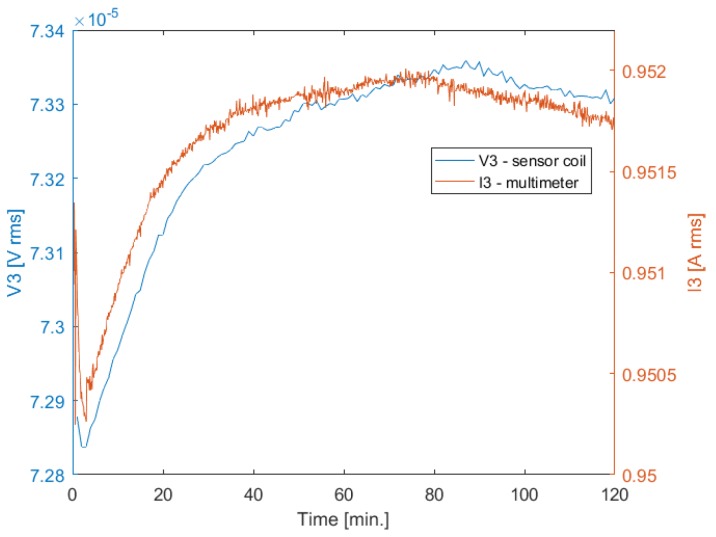
Voltage induced in the coil sensor and current measured with a multimeter—channel 3.

In Figure 5 we can observe that there is a correlation between the voltage drift of the sensor coil and the current drift of the field generator measured by the multimeter, which present similar behavior. We can therefore assume that the drift is due either to the Hall effect sensor, or the shunt resistor at its output, or both. In fact, the control loop varies the excitation current, in order to keep constant the current measured with the Hall effect sensor, which is affected by drift errors; hence, the measured current is stable, while the actual current drifts over time, causing a drift of the induced voltage.

In order to quantify and reduce the voltage drift, several experiments have been executed. In particular, five acquisitions have been performed, which differ in the choice of the regulated variable for the current control loop [Appendix A]. For each acquisition, the sensor has been sequentially placed in six positions by means of the robotic arm, at different distances from the FG, from about 250 mm to 750 mm along the *z*-axis. The voltage drift is higher when the sensor is closer to the FG, so it is important to quantify it as the distance varies. For each position, M = 100 measurements of the induced voltage have been performed, and the mean value was computed to reduce the noise. Each acquisition has been executed for a period of 120 minutes, starting after a zero warm-up time. The results have shown that the drift was approximately the same at all distances, in percentage terms. Moreover, the system performed better when choosing $I_{i}^{\prime \prime \prime }$ as regulated variable [Appendix A], because the voltage drift reached a stable value for all channels, within 0.03%, 0.50%, 0.10%, 0.25% and 0.01% of the final value, respectively, after a warm-up time of about 50 min.

## 5. Validation Tests

Validation tests were carried out to evaluate the performance of the proposed system in terms of the repeatability of the sensor position estimation. The results were obtained by means of the post-processing of the acquired data, and not in real-time.

### 5.1. Repeatability Error Evaluation

To evaluate the repeatability error, the standard deviation of repeated measurements of the sensor position $r={\left[x,y,z\right]}^{T}$ was calculated. The magnetic sensor measured the generated magnetic field, returning five rms components, denoted as $v={\left[v_{1},\ldots ,v_{5}\right]}^{T}$. These were affected by errors, assumed to be uncorrelated, with diagonal covariance

(2) $$C_{v}=\left[\begin{array}{ccc} \sigma_{v_{1}}^{2} & \ldots & 0\\ \vdots & \ddots & \vdots \\ 0 & \ldots & \sigma_{v_{5}}^{2} \end{array}\right]$$

The position standard deviation $\sigma_{r}={\left[\sigma_{x},\sigma_{y},\sigma_{z}\right]}^{T}$ can be calculated by the propagation of the voltage standard deviation $\sigma_{v}={\left[\sigma_{v_{1}},\ldots ,\sigma_{v_{5}}\right]}^{T}$, as follows.

Let $g={\left[g_{1},g_{2},g_{3},g_{4},g_{5}\right]}^{T}:Χ⫃R^{3}\to R^{5}$ be the function relating r and v:

(3) v=g(r)

Relation (3) is sampled experimentally by placing the sensor on a regular grid of $N_{p}$ points $r_{i}$, $i=1,\ldots ,N_{p}$, obtaining voltages $v_{i}$. 

The variation of g(r) in the neighborhood of a point $r_{i}$ can be evaluated by means of the gradients $\nabla g_{j}\left(r_{i}\right),$ defined as:

(4) $$\nabla g_{j}\left(r_{i}\right)=\left[\frac{\partial g_{j}}{\partial x}\left(r_{i}\right),\frac{\partial g_{j}}{\partial y}\left(r_{i}\right),\frac{\partial g_{j}}{\partial z}\left(r_{i}\right)\right];$$

$$j=1,\ldots ,5,i=1,\ldots ,N_{p}$$

The partial derivatives are estimated experimentally by the ratios $\frac{∆v_{j}}{∆x}$, $\frac{∆v_{j}}{∆y}$ and $\frac{∆v_{j}}{∆z}$, by assuming small variations in the sensor position. 

Then, the Jacobian matrix $J_{g}\left(r_{i}\right)\in R^{5ȕ3}$ can be calculated, defined as:

(5) $$J_{g}\left(r_{i}\right)=\left[\begin{array}{c} \begin{array}{c} \nabla g_{1}\left(r_{i}\right)\\ \ldots \end{array}\\ \nabla g_{5}\left(r_{i}\right) \end{array}\right].$$

To evaluate the relation between small changes of r and v, it is useful to linearize (3), obtaining

(6) $$v≃g\left(r_{i}\right)+J_{g}\left(r_{i}\right)\left(r-r_{i}\right)$$

We can invert (6) by means of the Moore–Penrose pseudoinverse of the Jacobian matrix, $J_{g}{}^{+}\left(r_{i}\right)\in R^{3\times 5}$, in order to estimate r from v:

(7) $$r≃r_{i}+J_{g}{}^{+}\left(r_{i}\right)\;\left(v-v_{i}\right).$$

By using (7), the position covariance $C_{r_{i}}$ can be calculated from the covariance of voltage components, $C_{v}$:

(8) $$C_{r_{i}}≃J_{g}{}^{+}C_{v_{i}}\;J_{g}{}^{+T}$$

Finally, the variance of position coordinates can be found on the diagonal of $C_{r_{i}}$, after substituting (2) into (8):

(9) $$\sigma_{r}{{}_{i}}^{\circ 2}≃J_{g}{}^{+}{\left(r_{i}\right)}^{\circ 2}\;\sigma_{v}{{}_{i}}^{\circ 2}$$

where ∘ is the Hadamard power operator, and ∘2 denotes the element-wise square. The square root of $\sigma_{r}{{}_{i}}^{\circ 2}$ obtained in (9) allows to estimate the standard deviation of each coordinate.

It should be noted that the obtained result can be applied to any position reconstruction algorithm, as long as it can be linearized for error propagation purposes.

The same method can be applied to estimate the effects of the residual drift of voltage measurements, by means of (7).

### 5.2. Test Protocol and Results

A suitable test protocol was defined to evaluate the system performance in a wide operating volume. 

Preliminary experiments were executed to evaluate the repeatability of the system for three different sensor orientations, with its axis aligned along the *x*-, *y*-, and *z*-axis of the reference system of Figure 2, evaluating the repeatability in several positions along the *z*-axis.

**Figure 6:**
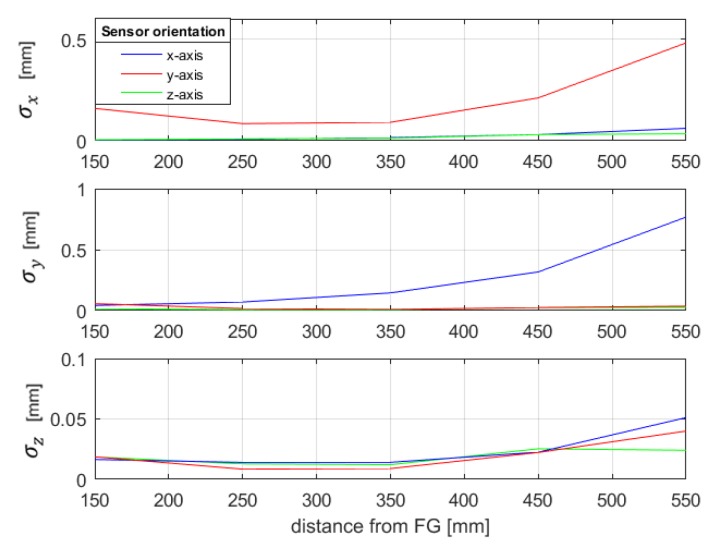
Position repeatability obtained with the estimation through gradients for different orientations of the sensor—EMTS prototype.

In particular, for each sensor orientation, the sensor has been placed in five locations along the *z*-axis, 100 mm apart, at a range of about 150 to 550 mm from the FG. For each position, 100 voltage measurements have been performed, and the mean and standard deviation have been computed. Moreover, for each position, the voltage gradients (4) have been calculated as finite differences between the mean voltage measured at $r_{i}$ and the ones measured at a small distance of 5 mm moving along the *x*-, *y*- or *z*-axis, separately. Then, we obtained five different Jacobian matrices and the correspondent components of the pseudoinverse matrices $J_{g}{}^{+}\left(r_{i}\right)$, for each sensor orientation. Afterwards, the position SD has been estimated by means of (9). The results are shown in Figure 6. It can be noted that the repeatability changes as the orientation varies.

In a subsequent analysis, the repeatability error of a single sensor orientation was studied at more positions over the range, to investigate the effect of the distance from the field generator on the position repeatability. In particular, a middle orientation has been chosen, so that the axis of the sensor formed an angle of −30° in the *xz*-plane; in this case, it was possible to approach the condition where the orientation provided worse repeatability.

**Figure 7:**
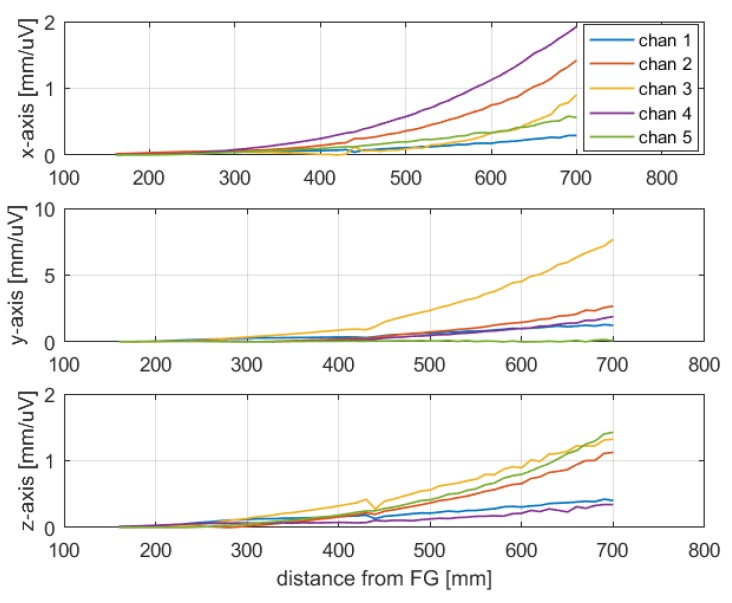
Elements of the pseudoinverse of the Jacobian matrix vs distance from the FG.

The sensor was placed in 55 different points along the *z*-axis, with a step of 10 mm, in the range of about 160 to 700 mm from the FG. The same test protocol of previous experimental tests was applied, thus obtaining 55 different Jacobian matrices and the corresponding components of the pseudoinverse matrices $J_{g}{}^{+}\left(r_{i}\right)$, shown in Figure 7. Basically, each component of $J_{g}{}^{+}\left(r_{i}\right)$ represents the system sensitivity of a position coordinate when each voltage component changes. 

**Figure 8:**
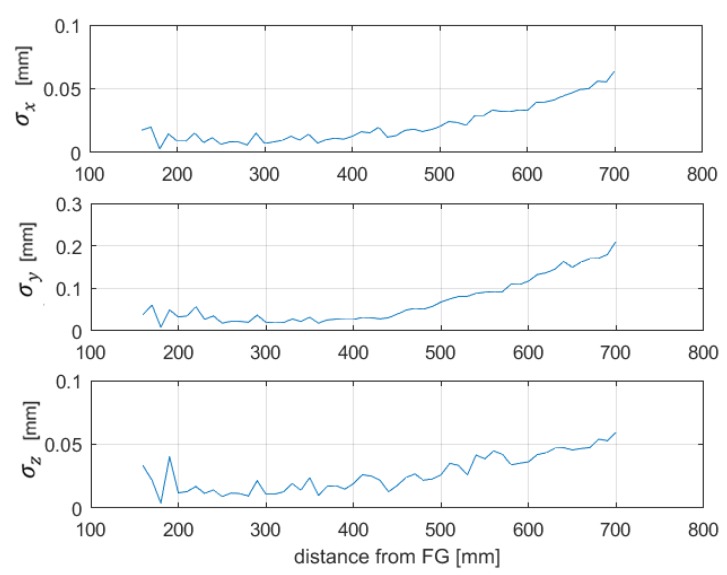
Position repeatability obtained with the estimation through gradients.

Afterwards, the position SD was estimated by means of (9). The test has been executed after a warm-up time of 1 hour. The obtained results, shown in Figure 8, show low position repeatability errors along the *x*-, *y*- and *z*-axis, of about 0.2 mm, within the limit suitable for surgical navigation, of about 1–2 mm in several applications [16,28]. Moreover, it can be noticed that the position standard deviation increased with the distance from the FG, as was expected, since the amplitude of the magnetic fields, the gradients and the signal to noise ratio of the sensor output were lower at a greater distance from the FG. This was also confirmed by the increasing behavior of the pseudoinverse observed in Figure 7. Indeed, its dependence on position allows one to evaluate, at different distances, the sensitivity of the position error to measurements errors on each channel. For example, channel 1 exhibited a lower sensitivity to errors overall. These considerations may be useful to assess the performance of the transmitting coil of the FG relevant to each channel, which is affected by its distance from the sensor coil.

**Figure 9:**
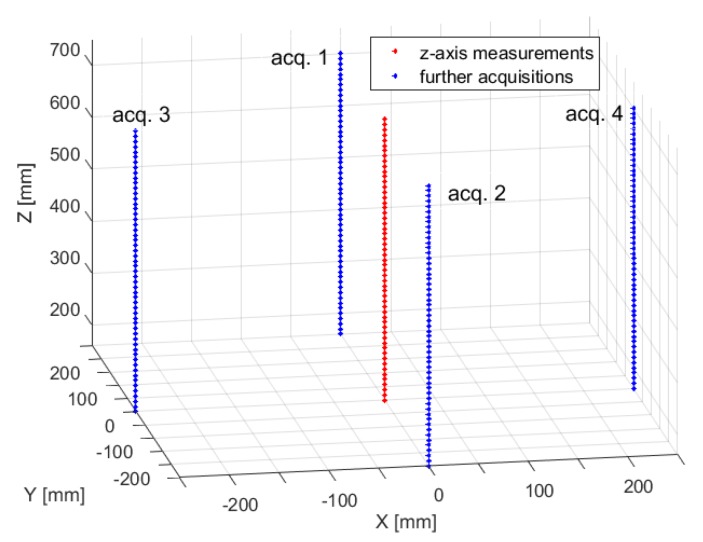
Sensor positions of four additional experimental tests. The red points represent the sensor position of first acquisition where all points lie on the *z*-axis.

Four additional experimental tests have been performed by considering further sensor positions to evaluate the position repeatability in a larger working volume, as shown in Figure 9. The obtained results are similar to the ones presented in Figure 8.

**Figure 10:**
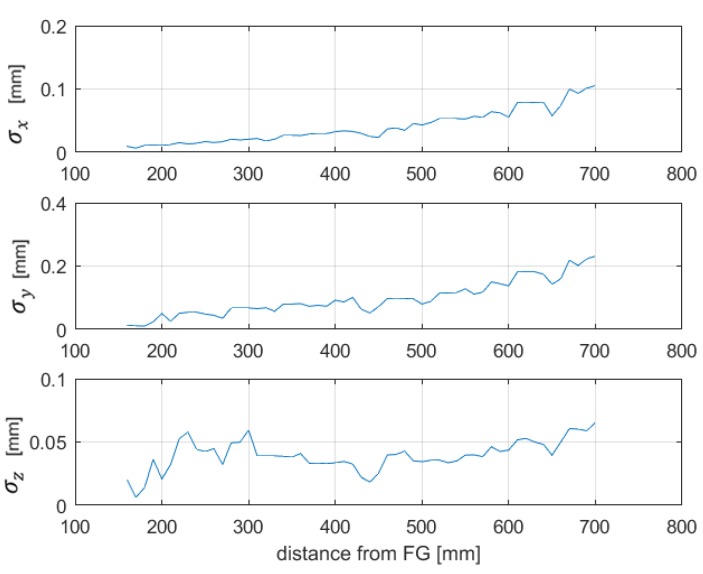
Position repeatability obtained with the estimation through gradients for the trajectory labeled “acq. 2”.

For example, Figure 10 shows the SD for (x,y)=(0,−250) mm, obtained by varying the *z* coordinate on the trajectory labeled “acq. 2” in Figure 9.

### 5.3. Analysis of Drift Effect

In order to quantify the effect of the drift on the system accuracy, we repeated, after 90 min, the experimental test described in Section 5.2, keeping the whole system turned on. Then, the voltage differences between the two sets of experiments, $∆v_{i}={\left[∆v_{1}{}_{i},\ldots ,∆v_{5}{}_{i}\right]}^{T}$, have been measured in each of the 58 points, for each channel, and the position errors $e_{r}{}_{i}={\left[e_{x}{}_{i},e_{y}{}_{i},e_{z}{}_{i}\right]}^{T}$ have been estimated by means of (7), where $J_{g}{}^{+}\left(r_{i}\right)$ are the same as those calculated in Section 5.2.

**Figure 11:**
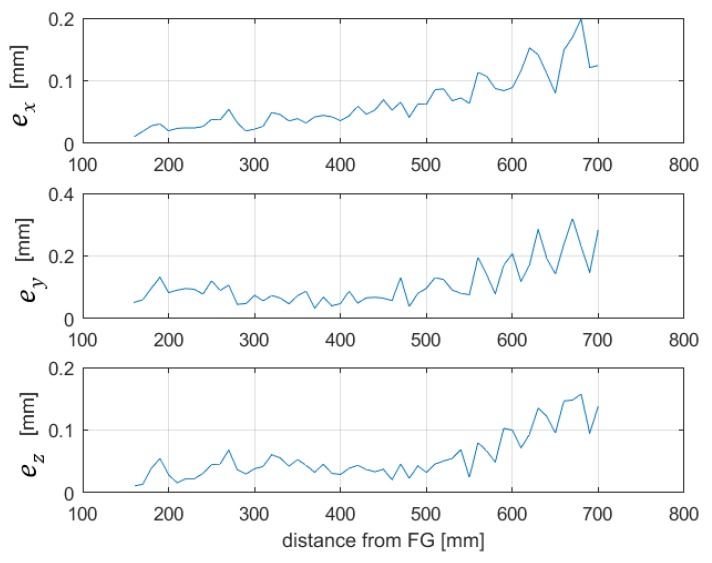
Position errors caused by drift, obtained by repeating the same measurements after 90 min and applying the estimation through gradients.

Figure 11 shows the position error contribution given by the residual drift of the voltage measurements and is the result of (7) and the values of the elements of the pseudoinverse of the Jacobian matrix, which are shown in Figure 7. These array elements represent the sensitivity of the position error and repeatability to the errors of the voltage components, such as drift and noise. It can be observed that the position errors are of the order of 0.3 mm, meeting the specifications required for surgical navigation in many applications [16,28].

### 5.4. Comparison with the State of the Art

Aurora systems (NDI) are the most used EMTSs in surgical navigation. To compare the repeatability error of the proposed system with the state of the art, the same experimental tests described in Section 5.2 have been performed by using the Aurora PFG 20-20 system [21]. This system consisted of: (i) a Planar Field Generator, (ii) a Sensor Interface Unit that amplifies and digitizes the electrical signals from the sensors, (iii) a System Control Unit that controls the FG, collects information from the Sensor Unit Interfaces, calculates the position and orientation of each sensor and interfaces with the host computer, (iv) a Five DoF (Degrees of Freedom) electromagnetic sensor.

**Figure 12:**
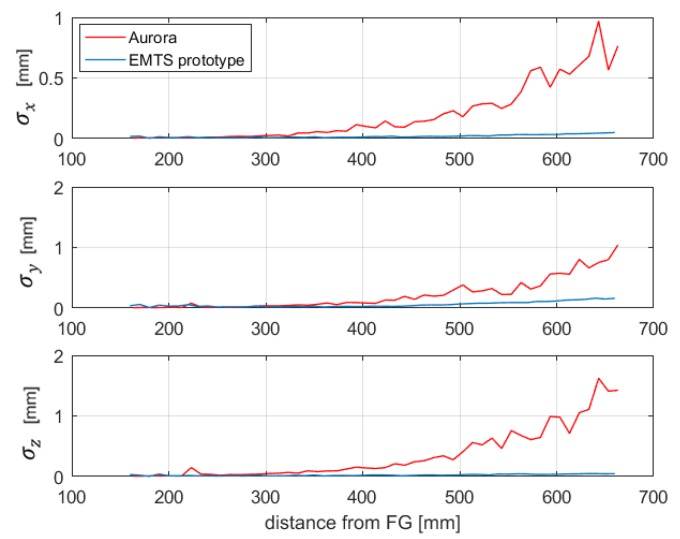
Comparison between the SD of the Aurora system and the EMTS prototype.

The Five DoF sensor has been placed in 51 points along the *z*-axis of the PFG 20-20, with a distance ranging from about 160 to 660 mm, which is the maximin distance from the FG indicated in the Aurora specifications. Figure 12 shows the SD of 100 measurements at each distance, obtained with both Aurora and the proposed system. It can be observed that the repeatability errors of the Aurora system are one order of magnitude greater than the ones obtained with the proposed system.

**Figure 13:**
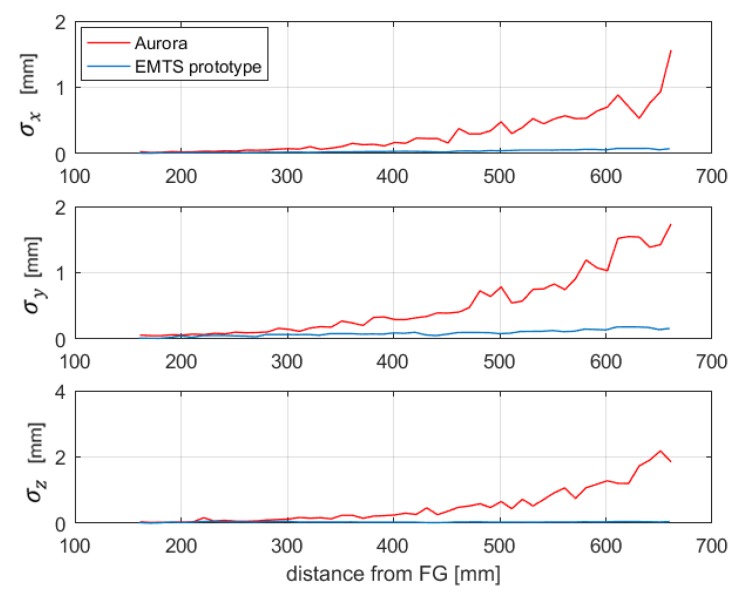
Comparison between the SD of the Aurora system and the EMTS prototype obtained along the trajectory “aq.2”.

Moreover, further measurements have been performed, according to the sensor positions indicated in Figure 9, to explore a larger volume. The results obtained in these new positions confirm the ones presented in Figure 11, as can be seen, for example, in Figure 13, which shows the repeatability on the trajectory labeled “acq. 2”.

## 6. Conclusions

In this paper we have illustrated a method to evaluate the repeatability of electromagnetic tracking systems, and we have applied it to a prototype that has been developed with the purpose of overcoming the limitation of current commercial systems, providing an accurate tracking of surgical tools far from the field generator.

Several hardware improvements have been described, which led to an enhanced system performance in terms of noise level reduction.

The repeatability error in sensor position estimation has been evaluated through the propagation of the voltage measurement error, at different distances from the FG, from about 160 mm to 700 mm, and the performances have been compared with results obtained from the tests executed on the Aurora system, obtaining SD values lower than 0.2 mm at 700 mm from the FG.

The analysis methodology used in this paper is based on the experimental measurement of field gradients, on the evaluation of sensor coil voltage noise and drift, and on the linear approximation of the voltage-position relation, which lead to an error propagation Formula (9). The proposed analysis has the advantage of relating the overall system performance to that of identifiable components which can be separately analyzed and optimized, on the one hand, and compared among different EMTSs, on the other. 

## Figures and Tables

**Table 1:** Relative SD of the induced voltage at 0.7 m and 1 m from FG, before and after hardware improvement, and their relative reduction.

	Distance (m)	*f*_1_	*f*_2_	*f*_3_	*f*_4_	*f*_5_
**Before improvement**	0.7	19.75%	0.15%	1.53%	0.08%	0.11%
	1.0	13.90%	0.40%	6.95%	0.19%	0.31%
**After improvement**	0.7	1.13%	0.04%	1.06%	0.03%	0.03%
	1.0	3.03%	0.13%	1.09%	0.10%	0.07%
**R** **e** **duction**	0.7	94%	73%	31%	62%	72%
	1.0	78%	68%	84%	47%	77%

## Appendix A

Let $I_{i}$ be the current in the *i*-th coil, and $I_{i,j}$ be the current in the *i*-th coil, filtered at the frequency $f_{j}$, j=1, …, 5.

Four different techniques to implement the control loop are defined, where the definition of the regulated variable changes each time [20].

(i) For the *i*-th coil, the regulated variable $I_{i}^{\prime }$ was evaluated by considering all the five harmonic components (j=1,…,5) present in the current measured on the *i*-th channel. Then, the following expression was used:
  $$I_{i}^{\prime }=\sqrt{\sum_{j=1}^{5}I_{i,j}{}^{2}}$$
(ii) For the *i*-th coil, the regulated variable $I_{i}^{\prime \prime }$ was evaluated by considering current components in the five coils at the same frequency *f_i_.* Then, the following expression was used:
  $$I_{i}^{\prime \prime }=\sqrt{\sum_{l=1}^{5}I_{l,i}{}^{2}}$$
(iii) For the *i*-th coil, only the current component at $f_{i}$ was considered.
  $$I_{i}^{\prime \prime \prime }=I_{i,i}$$
(iv) For the *i*-th coil, the measured current signal $I_{i}$ was used directly, without any filtering.
